# Challenges in preserving the “good doctor” norm: physicians' discourses on changes to the medical logic during the initial wave of the COVID-19 pandemic

**DOI:** 10.3389/fpsyg.2023.1083047

**Published:** 2023-06-08

**Authors:** Maria Härgestam, Maritha Jacobsson, Fredrik Bååthe, Emma Brulin

**Affiliations:** ^1^Department of Nursing, Umeå University, Umeå, Sweden; ^2^Department of Social Work, Uppsala University, Uppsala, Sweden; ^3^Institute of Stress Medicine, Gothenburg, Sweden; ^4^Institute of Health and Care Sciences, Sahlgrenska Academy, Gothenburg University, Gothenburg, Sweden; ^5^Institute for Studies of the Medical Profession, Oslo, Norway; ^6^Unit of Occupational Medicine, Institute of Environmental Medicine, Karolinska Institutet, Stockholm, Sweden

**Keywords:** COVID-19, medical logic, physician, discursive psychology, neo-institutional theory, healthcare, pandemic response

## Abstract

**Introduction:**

The COVID-19 pandemic was a tremendous challenge to the practice of modern medicine. In this study, we use neo-institutional theory to gain an in-depth understanding of how physicians in Sweden narrate how they position themselves as physicians when practicing modern medicine during the first wave of the pandemic. At focus is medical logic, which integrates rules and routines based on medical evidence, practical experience, and patient perspectives in clinical decision-making.

**Methods:**

To understand how physicians construct their versions of the pandemic and how it impacted the medical logic in which they practice, we analyzed the interviews from 28 physicians in Sweden by discursive psychology.

**Results:**

The interpretative repertoires showed how COVID-19 created an experience of knowledge vacuum in medical logic and how physicians dealt with clinical patient dilemmas. They had to find unorthodox ways to rebuild a sense of medical evidence while still being responsible for clinical decision-making for patients with critical care needs.

**Discussion:**

In the knowledge vacuum occurring during the first wave of COVID-19, physicians could not use their common medical knowledge nor rely on published evidence or their clinical judgment. They were thus challenged in their norm of being the “good doctor”. One practical implication of this research is that it provides a rich empirical account where physicians are allowed to mirror, make sense, and normalize their own individual and sometimes painful struggle to uphold the professional role and related medical responsibility in the early phases of the COVID-19 pandemic. It will be important to follow how the tremendous challenge of COVID-19 to medical logic plays out over time in the community of physicians. There are many dimensions to study, with sick leave, burnout, and attrition being some interesting areas.

## 1. Introduction

During a crisis, established routines must be changed and adapted to the prevailing situation. Crises are unexpected and characterized by uncertainty, which means that there is room for different interpretations and options for action (Schatzki, 2016). In other words, a crisis such as the COVID-19 pandemic was a natural experiment (Gross and Krohn, 2005; Gross, 2009), and knowledge of how to handle the acute situation that arose in the spring of 2020, especially in the Swedish healthcare, sector was poor (Nilsson et al., 2022). An earlier study shows that the organizational logic in Swedish healthcare changed when hospitals had to respond to the COVID-19 pandemic (Jacobsson et al., 2022). Furthermore, the challenges that physicians in Sweden faced in their working conditions during the pandemic's initial phase impacted their care provision experiences (Nilsson et al., 2022). In times when physicians can no longer trust their professional judgment and clinical expertise, they must instead find other ways to handle the medical responsibility of making good clinical decisions about immediate patient needs. This situation is often referred to as a situation of medical uncertainty (Han et al., 2011). Medical uncertainty can have aversive psychological effects on physicians, including thoughts and feelings of vulnerability, and can lead to a lack of decision-making and action. Physicians manage these effects and their experience of uncertainty itself through various strategies (Han et al., 2021), but the principal among these is the effort to seek information to reduce uncertainty. However, during the pandemic, no or very little information and knowledge existed (Nilsson et al., 2022). For physicians, there was a sense-making process when they had to interpret the encounter with the COVID-19 pandemic, a new condition that could not be understood and handled by the use of existing medical practices and guidelines (Weick, 1995; Weick et al., 2005). When new knowledge has to be created and established routines are no longer functional, this can be perceived as a disruption and something negative, but there can also be room for positive changes (Schatzki, 2016).

The norms of professional conduct for physicians include discourses of the good doctor, in which physicians have high-level evidence-based competence and professional judgment, balanced with great responsibility (Whitehead, 2011). Norms suggest that a “good doctor” uses both individual clinical expertise and the best available external evidence in clinical decision-making, and neither alone is enough (Sackett et al., 1996). The historical concept of the “good doctor” comprises a complex array of attributes and behaviors that physicians, already in medical school, learn to aim for (Whitehead, 2011). A recent review identified six different attributes that signify a “good doctor” (Steiner-Hofbauer et al., 2018). O'Donnabhain and Friedman (2018) list as many as 11 traits and seven behaviors of a “good doctor.” Based on these two publications, typical identity attributes building up the “good doctor” are strong interpersonal skills, communication, patient involvement and ethics (including being compassionate, empathic, a good listener, responsive, humane, and honest), leadership (i.e., motivates and supports colleagues, teaching and supervision, and persistent), and sound clinical decision-making (i.e., medical management, remain current with the medical knowledge and evidence base, and contributes to a scientific understanding of disease).

Modern medicine involves three pillars of knowledge that physicians, to fulfill the identity of the “good doctor,” need to integrate when making patient-care decisions: published evidence, clinical judgment, and the patient's values and preferences (Sackett et al., 1996). The focus on evidence in medicine is supposed to safeguard the patients and provide quality care, and practicing evidence-based medicine means integrating clinical expertise with the most recent clinical research in making decisions about the care of individual patients (Sackett et al., 1996). Being a “good doctor” underlies many physicians' view of their profession as a calling (Dzau et al., 2018). However, the COVID-19 pandemic has, by no doubt, been one of the most significant challenges to the practice of evidence-based medicine (Carley et al., 2020; Pacheco-Barrios and Fregni, 2020), impacting the foundation of being a “good doctor” at its very core (Pacheco-Barrios and Fregni, 2020). In this study, we make use of neo-institutional theory and discourse psychology to gain a more in-depth understanding of a situation when physicians are challenged in their profession and practicing modern medicine. In specific, the aim was to explore how physicians in Sweden narrate how they position themselves as physicians in relation to practicing modern medicine during the first wave of the COVID-19 pandemic. To the best of our knowledge, this is the first article with this approach.

### 1.1. Theoretical approach

In this study, our point of departure is the ***medical logic*** which we define as one part of the overall institutional logic in healthcare. Institutional logic is a concept used within the neo-institutional theory (Powell and DiMaggio, 2012) to visualize different spheres with different belief systems that maintain different types of relationships in and between organizations. We use this theory to get a deeper understanding of how physicians relate to organizational conditions when they have to carry out medical assessments. Logics are about the rules, routines, and values that give legitimacy, stability, and meaning to how individuals act and communicate within organizations.

Medical logic includes rules and routines combining research-based evidence with practical experience that condition clinical decision-making. Medical logic is foundational when it comes to physicians diagnosing, explaining, and treating the physical bodies of patients (cf. Rosenberg, 2007) and thus central in the discourse to form the concept of the “good doctor.”

Different circumstances in the healthcare institution condition physicians' clinical decisions. These circumstances are what Scott (1995) terms regulative, normative, and cognitive elements. These elements both structure and constrain behaviors in institutions, fostering the identity of the good doctor (Whitehead, 2011). *Regulatory* elements (must do) are laws and formal regulations, often formulated as clinical guidelines, that set the framework for the activities within the organization for the physicians. The regulatory elements give physicians a certain degree of autonomy in their work. They can, to a certain extent, act independently when it comes to medical decisions (Forsberg Kankkunen and Bejerot, 2017). In addition, according to regulations in Sweden, physicians can delegate some responsibilities to other professionals. The *normative elements* (should do) are more prescriptive and are based on standards, values, and norms that will guide members within the organization. In their clinical work, evidence-based medicine sets a range of normative elements of what physicians should do (Sackett et al., 1996) and for sound clinical management (O'Donnabhain and Friedman, 2018; Steiner-Hofbauer et al., 2018). *Cognitive elements* (want to do) are about cultures and routines that are taken for granted, the “common sense.” Physicians are taught already in medical school that common sense aligns with the norm of being the “good doctor” (Whitehead, 2011).

Normative and cognitive elements scaffold individuals in organizations to pursue a learned, correct socialized behavior. The regulatory elements provide yet another firmer structure intended to regulate and limit more extreme versions of “incorrect” behaviors. Each of these elements draws on one or more sources of legitimacy by being legally sanctioned, morally authorized, and culturally supported. When regulative elements are weak, normative and cognitive elements change (Jacobsson et al., 2022).

To better understand how physicians construct their versions of what happened during the pandemic and how it affected their medical logic, we are inspired by discursive psychology. Discursive psychology is both a theoretical orientation and a methodological approach when it comes to studying language as a medium of human action (Potter, 2012). With the help of discursive psychology, we can capture how physicians, with the help of language, take certain positions in relation to organizational conditions. Language is not considered a mirror of the real world; language creates particular versions of the world and is situated in a given context. The language will be analyzed from a micro perspective but will be interpreted from a broader macro perspective since it is linked to ideologies, cultures, and contexts (Wetherell, 1998).

To find out how the physicians made sense of changes to the medical logic and how the discourses of a good doctor were challenged during the initial wave of the COVID-19 pandemic, we identify ***interpretative repertoires*** in the interview material. Interpretative repertoires refer to “recurrently used systems of terms used for characterizing and evaluating actions, events, and other phenomena” (Potter and Wetherell, 1987) (p. 149). The interpretative repertoires provide actors with different subject positions. The subject position is defined as the individual's “location within a conversation” (Edley, 2001), which means that positions are adopted and become relevant within a specific conversation. Wetherell (1998) emphasizes the individual's multiple positions and the possibility of showing the variety of available subject positions that are negotiated in talk and interaction. Parts of previous positions persist in the current situation and could be seen as a sedimentation of past discursive practices (Potter and Wetherell, 1987). The individual can vary positions within a conversation as well as between conversations, which means that they both produce and are a product of different repertoires. When individuals choose possible, preferable, rhetorically effective, or available repertoires, the subject position is *untroubled* (Wetherell, 1998; Staunæs, 2003). When individuals are using repertoires that are not interpreted as preferable, by themselves or by others—the position is *troubled* (ibid).

When individuals end up in troubled positions, ideological dilemmas can arise. Billig et al. (1988) used the concept of “***ideological dilemma***.” According to Billig et al. (1988), ideology can be described as “common sense” in a specific time and context. Ideological dilemmas are embedded in different forms of knowledge. Scientific knowledge and scientifically trained expertise have high value and are guarantors for facts and evidence in medical contexts, alongside experienced-based knowledge based on long clinical experience. This can produce a dilemma between competing types of knowledge. Billig et al. (1988) argued that a dominant culture exists within each community, consisting of authorities and experts that have been approved by society. In the medical context, the doctors' voices as experts are strong.

In this study, we analyze how physicians talk about their experiences of the COVID-19 pandemic in terms of medical logic. The overall aim was to explore how medical logic was challenged during the first response to the pandemic. The interviews were conducted during the summer after the first wave of the COVID-19 pandemic. We believe that it is important to capture the experiences that the physicians had during the initial and ongoing crisis. These initial reflections can be critical since significant insights may be lost if interviews are conducted in retrospect.

## 2. Materials and methods

This study applies a qualitative research design using neo-institutional theory and discursive psychology to gain in-depth knowledge of Swedish physicians' experiences working during the COVID-19 pandemic. This study gained ethics approval from the Swedish Ethical Review Authority (2020-02433). All participants gave their consent to participate both verbally and written.

### 2.1. Interviews

Invitations to participate in the study were advertised on social media and in the journal for physicians in Sweden. Those interested contacted the research team and were sent a more extended invitation with a description of the project and information about consent. All those who were initially contacted by the researchers also consented to be interviewed. Most (n=24) interviews took place in virtual meeting rooms and five in a location chosen by the interviewed physician. Data were collected between June and November 2020 by two authors (EH and FB). A semi-structured interview guide was designed using discussion themes, supportive questions, and probes. Themes were derived from previous research on psychosocial working conditions, physician wellbeing, and management and change in healthcare systems. The interview guide was tested in pilot interviews, and minor changes were made before the rest of the interviews were conducted. The discussion themes in the guide concerned experiences from the transition from regular care to pandemic care, leadership and organization during the transition, a normal day during the pandemic, patient care and quality of care, existential health and moral stress, work, and private life and the future (see Supplementary material for the full interview guide). All participating physicians were asked the same supportive questions while the probes differed depending on the experiences of the physicians and their willingness to talk.

Due to early reports from Italy and China that healthcare professionals working with patients infected with COVID-19 showed symptoms of post-traumatic stress disease (PTSD), each interview proceeded with initial questions screening for PTSD. None of the participating physicians showed clear symptoms of PTSD, and interviews could proceed. Interviews took between 60 and 90 min and were audio-recorded and transcribed verbatim by an external part.

### 2.2. Participants

A total of 28 hospital-based physicians were interviewed. The physicians worked in different geographical locations and regions in Sweden. Their experience as a physician ranged from 8 to 27 years. In total, five were consultants, 12 were attending physicians, and 11 were resident physicians. They were specialists or under specialist training in internal medicine (including infectious diseases), neurology, orthopedics, pediatrics, and anesthesiology. In total, 17 of the interviewed physicians were women, 15 were living with a partner and had children, two were living alone with shared custody of children, and two were single with no children.

### 2.3. Data analysis

In reading and analyzing the empirical material, we identified interpretative repertoires within medical logic. In the analysis, we searched for patterns in the empirical material based on subject positions and interpretative repertoires (Potter and Wetherell, 1987; Wetherell, 1998; Staunæs, 2003). The analysis process was led by authors MH and MJ. All four authors regularly met to discuss the analysis and results throughout the analysis process.

The analyzing process began with a close reading of transcribed interviews. The coding was initially inductive and descriptive. After that, occurring themes or ways of talking were identified. Keywords and recurring themes were grouped with an interpretive approach to gain into what is being said and *how* it was said (Seymour-Smith, 2017), which means that we were looking to identify how the physicians articulated their understanding of *if* and, in that case, *how* their thoughts on medical decision-making changed during the initial wave of the COVID-19 pandemic. To study *how* the interviewed physicians verbally constructed their versions of what happened during the pandemic, we initially analyzed three of the interviews more thoroughly with central concepts from discursive psychology and neo-institutional theory which was discussed between authors. Subsequently, interpretive repertoires were identified by, in more detail, studying discursive constructions in relation to subject positions (Wetherell, 1998) and ideological dilemmas (Billig et al., 1988). An interpretive repertoire can be described as a recognizable way of describing, framing, or talking about a phenomenon (Potter and Wetherell, 1987). Thus, the full research process was abductive, which means combining induction and deduction and altering between empirically studying the material and theoretically analyzing (Dubois and Gadde, 2002).

## 3. Results

The result shows that the interviewed physicians faced extremely challenging situations during the initial wave of the COVID-19 pandemic. They were challenged with an unknown disease with symptoms among patients who did not follow traditional utterances, leaving them without research-based medical evidence and without knowledge from practical clinical experience. This left the physicians without clinical guidelines, structured rules, and routines to support their clinical decision-making about how to treat COVID-19-infected patients best. On top of that, COVID-19 was an unclear yet highly infectious virus, and the supply of personal protective equipment (PPE) was limited. In addition, the interaction and communication with the patient and their relatives were negatively impacted.

Overall, in the initial phase and throughout the first wave of the pandemic, a knowledge vacuum occurred (Jacobsson et al., 2022) that deeply challenged physicians' medical logic. The three knowledge pillars of modern medicine, published evidence, clinical judgment, and patient communication (Sackett et al., 1996) were all impacted, challenging the possibility of acting in line with what is expected of a good doctor.

In our analysis, we have identified four interpretative repertoires: *medical evidence, clinical judgment and prioritization, patient communication*, and *risk*. In these repertoires, the physicians talked about factors related to regulative, normative, and cognitive elements that affected their decisions and behaviors and how their positions as physicians changed during the pandemic.

### 3.1. The repertoire of medical evidence

The repertoire of medical evidence illustrates the vacuum that arose in the lack of regulative elements and having no evidence-based knowledge. The physicians described the symptoms of the COVID-19 virus as unfamiliar. They could not use their current knowledge to safeguard and treat the patients since patients reacted in unpredictable ways. Since there was no, or limited information from traditional and formal channels, such as the hospital management or scientific guideline committees, other sources of information became important. Colleagues at different hospitals and/or in other countries that could contribute with updated information on social media became important.

The COVID-19 virus behaved in other ways compared to previous SARS viruses. Patients infected had unrecognized symptoms and responded to traditional treatments in a non-traditional way. The state of knowledge changed rapidly, and there was a clinical need to be updated several times a day. In the initial stage, there were no clear and stable clinical guidelines on how patients should be treated. The treatment strategy in the morning was sometimes out of date in the afternoon (IP6), and according to the interviews, this created a feeling of an experimental treatment for this “unknown” disease. At first sight, the patients seemed to be well; they were texting their relatives on their phones, but suddenly, in the next moment, they collapsed.

“*And when I actually got scared, that was when you started to realize that these patients could have neurological problems, and we had a patient lying with seizures, and the neurologist was there, and they told us, but we have just had our first patient with haemorrhagic encephalopathy, so some kind of general bleeding brain and then it became like this ohh I do not want to hear this, I thought this was a respiratory infection*.” (IP 14)

In the excerpt above, the physician described a medical dilemma. The symptoms of the patients with a suspected COVID-19 infection did not show the expected symptoms of a patient with respiratory disease (IP6). More suspicious was that despite oxygen treatment, patients did not improve. However, COVID-19 turned out to affect not only the patient's respiratory but also neurological symptoms such as seizures that later turn out to be a result of a brain hemorrhage (IP14). Informants described how their positions changed and that they became more dependent on support from colleagues. At the clinics, daily physical meetings, formal and informal discussions, and seminars, continuous updates on the state of the pandemic contributed with support in complex cases. As the patients showed new severe and extraordinary symptoms, informal networks with colleagues provided vital knowledge and support.

“*So that helps, it makes you feel not so lonely, and you do not feel alone when you meet your colleagues, but even when you cannot, it probably feels like you know that you are not alone. Then if there is a particular decision that is tricky or so, but it would be exceptional, you can still ask many, and then you will not be alone about it either*.” (IP16)

In the excerpt above, the interviewed physicians emphasized the problems with the position of being “alone” several times. For the interviewed physicians, social media (chatrooms and face-to-face conversations) became an important platform not only for providing knowledge and updated information about COVID-19 but also for establishing formal and informal networks with colleagues, both nationally and internationally. Earlier research has shown that online groups help people to improve their psychological wellbeing during the COVID-19 crisis (Marmarosh et al., 2020). The physicians described how these informal groups offered an opportunity to discuss the pandemic and exchanged experiences of how their work around the patients was organized and that it was important to belong to a group to find support in the knowledge vacuum.

The repertoire of medical evidence expressed by the interviewees shows that the medical logic changed during the pandemic. Since there was no or little empirical research and regulative elements, they could not lean on relating to what they must do. They had to find new informal groups where they could discuss medical decision-making in relation to normative and cognitive elements, what they should do, and what they wanted to do. The lack of knowledge and guidelines created dilemmas about what treatments to use for certain patients, which created conflicts between colleagues.

“*And then yes, as I said, not to be allowed to give, not to be allowed to try even with antibiotics when you want to, and I do not know, it may not be ethical, but it is, for me, it was, not to be allowed to try a treatment that might have worked and that was not as expensive as... It was not like rocket science.”* (IP 18)

### 3.2. The clinical judgment and prioritization repertoire

In the *clinical judgment and prioritization* repertoire, the interviewees described how they had to manage appropriate and safe care for many patients. A large number of seriously ill patients needed care, and it became clear that the capacity would not be enough. A big dilemma occurred when existing resources had to be prioritized. At the hospitals and care facilities, a discussion between physicians was initiated concerning treatment limitations. The interviewed physicians described that the preliminary statistics had shown excessive mortality among the patient group aged over 70. This created feelings of concern for physicians since this knowledge influenced how the resources such as medicines (IP 18) and visits to clinics (IP7) were prioritized. Before COVID-19, the healthcare system had no such restrictions, and this new experience created a feeling of “I could have done more.”

“*To not get, yes partly with this prioritization of place, that you leave a place empty just in case there might be someone who will need that place better, it was disgusting anyway.”* (IP18)

There occurred an ambiguity about how the separation of the patients would take place. For the patients who had respiratory symptoms, it was obvious that they should be isolated. However, patients with no symptoms ended up in regular wards where routines and guidelines on PPE were not as obvious, so there were some descriptions where both patients and personnel were infected by COVID-19.

“*We got corona to a department probably through staff. But it could just as easily have been some patient that we had and then moved from the admissions department, and the tests are not 100%, so above all, it is about sampling technique and how deleterious it would be if you missed such a case, that it is then added a corona patient into another department and then spreads. I think we had four deaths linked at least to one where it was spread on a regular department, so to speak. And that fear and anxiety, it was really hard, in fact, psychologically hard for oneself*.” (IP15)

Clinical judgment and prioritization were also affected by the lack of personal protective equipment (PPE). PPE had to be prioritized between the departments and personnel. Since the PPE was limited and they only could visit patient rooms, when necessary, nurses and physicians coordinated their tasks. This resulted in physicians doing nurses' work tasks and nurses doing physicians' work tasks if possible. The physicians described these changed positions as challenging but also developing. Physicians and nurses supported each other and moved across their safety zones, not in a dangerous way, but more as a helpful collaboration. (IP28). In the interviews, the physicians experienced this teamwork as positive and contributed to better communication between professions.

“*But we have a good structure, so we have tried to help each other, the physicians, the assistant nurses, and the nurses, we have tried as well. You cannot go in [to the patient] as many as you like, as often as you like, so we have, as it were, do each other's tasks with more or less success sometimes. When you as a physician go and have to make your assessment, and then you have taken the food tray, done the checks, tried to put some intravenous needle that you have not done in 20 years, it went very badly, so we have tried to help each other as well. And it's because when someone goes in [to the patient], we have to do as much as possible right then, so many parts have become very positive in our teamwork here as well. We help each other, and we move across our comfort zones but not in a dangerous way but more in a helpful way as well*.” (IP28)

There was not only a shortage of PPE but also a lack of critical medicines such as oxygen, antibiotics, and medical equipment such as hoses to ventilators in the ICU. This meant that the treatment strategies needed to be re-evaluated and re-prioritized. The lack of drugs could lead to unorthodox treatments; for example, in the ICU, anesthetic gases were used as sedatives instead of regular intravenous medicine (IP23). Lack of medicine, oxygen, and beds in the ICU challenged the normal procedures of safe and quality-secured medical management of patients.

“*For me, it is probably most important to tell this damn feeling when you could not help and did not get [to help] and then that you had to, that some, I had, these two specific, these patients who did not get the chance in the respirator and then… these two [patients] that I wanted to try antibiotics and did not get to do so and so, this feeling of not doing, I opt out of patients, that's it, it's like how hard it was and that the decision was not mine. But there are probably many who have experienced the same thing; I do not think I am alone in this*.” (IP18)

The proportion of seriously ill patients who sought care was more significant than the healthcare system had previously experienced. However, the already limited resources were not enough, and the lack of medication, equipment, beds, and personnel made it impossible to provide care as they had done a few months earlier. Instead, the interviewees describe how they had to negotiate with colleagues to prioritize resources between the patients. This repertoire also shows how the physicians and other personnel changed positions, helped each other, and tried in conversation with each other to expand the normative elements agreeing on what they should do to provide the best care for the patients in their clinical work.

### 3.3. The patient communication repertoire

The patient communication repertoire was about how the interviewed physicians experienced changes in relation to the patient, not being able to use the usual behaviors to interact effectively and ethically with patients and their relatives. The strict visiting restrictions at the hospitals led to reduced meetings between physicians and their patients, and visits from non-infected patients with non-emergency situations were canceled. According to the interviewees, they were prompted to book appointments by telephone or digital appointments, although, in some cases, this was not possible. Many of the patients belonged to vulnerable groups that had difficulty communicating, for example, patients with dementia and neurological diseases. Communication was also hampered by the fact that digitalization in healthcare had not been well developed and prioritized. Many physicians did not have the necessary equipment to have digital appointments (IP2). The canceled meetings affected the patients who were dependent on regular contact with the treating physician for adjustment of ongoing medication.

“…*the kind of questions you want to ask your Parkinson's nurse, you want to tell that now it has gotten worse, or you have problems with increased symptoms or you wonder what to do with a caring-related problem or what to do if something gets worse or when you get side effects. Those questions were delayed or unanswered*.” (IP4)

Established communication channels between healthcare professionals and patients in physical meetings did not work, and the interviewed physicians were worried that the patients would not receive the help they needed. They also expressed that there were communication problems with patients in the clinics since the communication was constrained due to the PPE as visors and face masks. When wearing face masks, many of the patients were not able to hear what the physician said, and this led to many misunderstandings. In addition, for those patients who did not speak or understand Swedish, it became even more complicated to understand as no relatives or interpreters were allowed to attend to explain and translate.

“*We especially had one [patient] that I remember, I worked at infection [department], a man from Somalia with mild dementia and did not understand any Swedish and did not understand anything, so he did not understand, he was very seriously ill and then with this mild dementia basically and so not know any Swedish. You could see the horror shine in his eyes, and it was so awful, and so I had to call his daughters and say please, please you cannot come, no you cannot come here, maybe if he gets much worse so that we think he will not make it, then maybe one of you may come, but not all may come*.” (IP 14)

Another communication problem in relation to patients was the physicians' contact with relatives. Due to the restricted visiting policy, the relatives were not allowed to visit their seriously ill and dying family members. This was very difficult for relatives to accept, and many of them reacted with anger. One example of a troublesome situation that came up in an interview was when a family had been notified that the prognosis for their family member was pessimistic. According to the existing restrictions, the physician had to refuse the relatives to visit.

“*...and say it to the patient, of course, this will go well, but I still think you should take the opportunity to call and talk to your wife. Okay, what do you really mean? Should I say goodbye to my wife because it will not work, or should I listen to you, it will go well?”* (IP 23)

The physicians describe how difficult it was to argue in favor of the restrictions, not allowing relatives to come to the hospitals and visit the patients (IP 28). Moreover, communication with relatives that regularly occur at the bedside had to be moved to telephones. Physicians spent a lot of time describing the situation of the patient to their relatives.

In the patient communication repertoire, the physicians described ideological dilemmas that ethically occurred. They came in troubled positions and had difficulty finding other, new, and good ways to communicate with the patients and relatives, given the restrictions.

### 3.4. The risk repertoire

The risk repertoire concerns how the physicians experience a threat to health and wellbeing. The risk included the patients' lives, their own and their colleagues' lives, and also the risk that they would infect their relatives. The risk repertoire also concerns the unpleasant situations physicians faced when they had disagreements with colleagues (IP 10) and/or the management (IP3).

The interviewees gave several examples of when they were worried about the risk of being infected with COVID-19. One example was when a colleague became seriously ill and died following a COVID-19 infection (IP3). Another example was when a COVID-19-infected colleague had complications with diffuse symptoms and long-term sick leave (IP17). The situation was expressed by the interviewee to be out of control. One of them said that she questioned her work and was even considering quitting her current post as a physician. The realization that healthcare did not act as expected created an identity crisis about being a physician.

“*And we have always felt that Sweden is an incredibly good place to be in if you are not if you are such a dutiful person, and now the whole world has collapsed for both my husband and me, really this whole bubble has just burst, there is nothing. I cannot trust my colleagues; I cannot trust that the health care will take care of me because they have not really done that, they had not taken care of me when I was sick, they have not wanted to take me now, I still have symptoms, it's like… it's the biggest crisis of my life*.” (IP17)

In the excerpt above, the interviewee uses an extreme case formulation (Pomerantz, 1986): the “biggest crisis of my life” to emphasize how COVID-19 has changed her life and her view on healthcare. The repertoire of risks was both concerned with becoming infected but also about “bringing” the infection home to the family. There are descriptions of how the physicians organized special arrangements with separate places to live to avoid exposing the family to the risk of being infected (IP 9). They looked, for example, into their life insurance (IP14). One of the interviewees married her spouse to secure the future of the family (IP 9). Another expressed that one of the hardest issues in his family life was that he had suddenly difficulty focusing on his children and being a part of their activities (IP1).

“*I was afraid that I would unknowingly have Covid or be mildly ill and pass it on to someone else; I was very worried about that. So, I tested myself many times before I got it; you could say, out of that fear, I have small children at home*.” (IP15)

The interviewees talked about their workdays as overwhelming with a stressful and chaotic clinical situation, with many departments overfilled with patients. The physicians noted an increased risk of missing important changes in the patient's status and treatment when there was limited time to document correctly (IP2). With the extraordinary work situation, many expressed concerns about how to handle the workload and long working hours. Many of the interviewees described how the intense work situation made it difficult to unwind when getting home and that they had sleeping problems and nightmares. Sometimes, the interviewed physicians had patients on their minds when they came home. In some cases, they were worried that the patient they had met could have been treated differently and perhaps survived (IP28). One of them expressed it as follows:

“*But this woman was not cared for where they usually care for that type of condition, either at the surgery department or the gastrointestinal department, but was cared for in isolation at infection (department) because we did not know if it was possibly a COVID infection and this woman passed away. And it's probably one of them; you asked me if I have had sleeping disorders, that is a patient that has been recurring in my mind because it was a very sad ending for that patient…....so, she died alone in the room because our staff was occupied, we did not have the opportunity to be in the rooms with these seriously ill patients. So, this is a patient who has followed me a little in my mind, and the relatives have for very obvious reasons been very sad and disappointed*.” (IP28)

The interviewed physicians described several situations where conflicts arose because they had different opinions or did not want to work in COVID-19 departments. Some did not dare to say no to volunteering to work at the COVID-19 department because of the risks that it could provoke colleagues and lead to conflicts. One physician described choosing to remain at her department to wait for further instructions from the closest leader. One of her colleagues was provoked by the fact that the physician did not volunteer to help at the COVID-19 department and started to yell and scream (IP10). Another of the physicians described how expressing conflicting views on principles for sampling for COVID-19 in patients led to threats on social media and aggressive e-mails from colleagues, creating a completely unexpected work situation.

“*I feel that no one listens at work, so I wrote on, you know that there is a physician-Facebook group and asked what it looks like in other Regions if, for example, you test people who have already had COVID-19, which [home Region] will not try, they have said no here. And at 11:30 p.m. I got threatening e-mails, yes, from colleagues at my clinic; it's true; I have saved everyone, taken screenshots, and so on. I have not talked to anyone except my husband, but yes, since then, I have not slept very well, I can say, and I go to work with a lump in my stomach and think, why I am here. If no one wants to listen to the facts, if no one even wants to discuss that maybe someone else has a different opinion, I may have, I'm wrong, I may not be right, but no one wants to discuss, but this is how the authorities have decided this, and you just have to keep quiet. And that is, it is very new to me, it was completely unexpected, it was unpredictable*.” (IP17)

Conflicts also arose between colleagues from different clinics from disagreements about prioritizing patients. The disagreements between physicians from different clinics were often related to when the patients from a specific department with COVID-19 needed to be isolated.

The need to belong is strong in humans, and therefore, the risk of not belonging becomes a serious risk. The interviewees narrated that expressing a different opinion concerning chosen treatment strategies was compatible with the risk of ending up on the “outside” of the group. One interpretation of this is that the physicians came in ideological dilemmas. Should they position themselves in untroubled positions in relation to the group and say nothing, or should they follow their assessment and put themselves in troubled positions in relation to the group?

“*And then I think that it is my conclusion now then after so many months that there is an incredible fear of conflicts, you must absolutely not contradict because then you have to argue for your cause and maybe you are the troublemaker or like… yes you want to belong to a group, you want to belong to*.” (IP17)

In the risk repertoire, the ideological dilemma was expressed as a risk for the patients' lives as well as their own and colleagues' lives, including the risk that they also might infect their families. In the analysis of the empirical material, fear of the unknown and losing control seemed to be important where disagreements and conflicts with colleagues were present.

## 4. Discussion

In this study, the empirical material resulting from interviews with 28 Swedish physicians was analyzed using neo-institutional theory and discursive psychology. The overall aim was to explore how medical logic was challenged during the first wave of the COVID-19 pandemic. The analysis resulted in four interpretative repertoires: medical evidence, clinical judgment and prioritization, patient communication, and risk. In the physicians' narratives, it appears that they experienced major identity challenges when key attributes of the “good doctor,” clinical knowledge, as well as evidence-based knowledge, were no longer available. The COVID-19 virus and the symptoms patients with COVID-19 presented with did not respond to the established clinical “common sense.” Thus, without their professional identity foundations available, i.e., medical evidence, clinical judgment, and patient communication domain (Sackett et al., 1996), a knowledge vacuum was created. In this knowledge vacuum, the interviewed 'physicians' regulative, normative, and cognitive elements changed, creating medical uncertainty (Han et al., 2011). The repertoires used by the interviewees showed how they were dealing with dilemmas that arose and that they had to change positions as physicians to deal with these unexpected crises and related uncertainties. The change in position challenged them in relation to the norm of being a “good doctor.” Clinical judgment and prioritizing are essential aspects of being a physician. The decision should be made based on both individual clinical expertise and the best available external medical evidence (Sackett et al., 1996). The main finding in this study was the vacuum that arose as physicians could not use their well-established medical logic and that they could not lean on existing regulatory and normative elements. In this vacuum, physicians still were responsible for clinical decision-making without a solid evidence base to fall back on. This knowledge vacuum challenged core attributes in the identity of being a “good doctor.” When identities are being challenged, strong emotions can be excited (Bååthe and Norbäck, 2013). Wright et al. (2017) draw attention to emotions and affective mechanisms in the processes of institutional work.

From the interviews, it was clear that no guidelines were available, and patients did not respond as expected when treated with help from previous experiences. The lack of guidelines posed stress to physicians and changes to the organizational logic (Jacobsson et al., 2022; Nilsson et al., 2022), contributing to the knowledge vacuum. These results align with the findings by Pacheco-Barrios and Fregni (2020), who suggested that the COVID-19 pandemic posed a tremendous challenge to the foundation of being a good doctor.

In the medical logic, patient safety and quality care were also disrupted, which brought moral stress to the physicians. At least in the initial wave of the pandemic, Pacheco-Barrios and Fregni (2020) suggest it also caused “patients” harm. The medical code of ethics also clashed with the need to prioritize certain patients for treatments and ICU care. The shortage of medicines, such as oxygen and antibiotics, and beds indicates that the physicians had to prioritize treatment for those patients who were estimated to survive a tough treatment and then rehabilitation. Physicians were not able to apply their interpersonal skills, and communication with patients and relatives was disrupted. As suggested by Carley et al. (2020), evidence-based medicine was challenged by the COVID-19 pandemic. However, in the interviews, physicians described how they, from their troubled positions, became active to find their own (new) solutions to rebuild a sense of medical evidence. For instance, groups on social media were a great unorthodox source of knowledge. Social media has been used by physicians from other countries as a source of current knowledge of the best practices for COVID-infected patients (Shekar and Aravantagi, 2020). Connecting with other physicians through the use of social media also contributed to an experience of not being alone. This finding corroborates previous research finding that social belonging has a positive correlation with wellbeing (Salles et al., 2019). This innovative way, and physicians becoming active in finding new ways out from the troubled position, resonates with Pratt et al. (2006), who concluded that identity construction is triggered by an experienced mismatch between what physicians did and who they strove to be.

In conclusion, during the first wave of the COVID-19 pandemic, a knowledge vacuum arose among physicians. In this vacuum, physicians could not use their common medical knowledge nor rely on published evidence or their clinical judgment. They were thus challenged as the “good doctor.” It will be important to follow how the tremendous challenge of COVID-19 to medical logic plays out over time in the community of physicians. There are many dimensions to study, with sick leave, burnout, and attrition being some interesting areas.

### 4.1. Implications

One practical implication of this research was to provide a rich empirical account where physicians are allowed to mirror, make sense, and normalize their own individual and sometimes painful struggle to uphold the professional role and related medical responsibility in the early phases of the COVID-19 pandemic. As authors, we hope this research can contribute to physicians noticing that one's own early COVID-19 experiences were shared and reasonable, given the odd situation with “a vacuum of knowledge.” Maybe this can contribute toward creating a sense of normalizing and belonging. This empirical research can possibly contribute toward healing invisible yet painful wounds that individual physicians can have received during the early phases of COVID-19 when upholding the professional identity of “a good doctor” was severely challenged. Indeed, this knowledge is also important for HR and managers in their essential task of taking care of the care providers so that the providers can take care of the patients (Bodenheimer and Sinsky, 2014). For future pandemics with high impact on healthcare, healthcare organizations need to support physicians through, for instance, forums where ethical and moral dilemmas and medical evidence can be discussed.

### 4.2. Strengths and limitations

In qualitative research, the purpose was not to extend findings derived from selected samples to people at large but rather to transform and apply the findings to similar situations in similar contexts (Polit and Beck, 2004). A strength of this article was that we analyzed material from 28 physicians with various specialities who, during the COVID-19 pandemic, worked in hospitals in different geographical areas in Sweden. This sample of physicians provides insights and reflections on the types of dilemmas and priorities faced during the pandemic.

In this study, we use discourse analysis on a microlevel, which provides a nuanced view of institutional processes. It is a method to study socially constructed ideas that underlie institutions and to question macro-institutional goals. We accept that any interpretation is one of many possible interpretations, but the findings in this article should be understood as relevant to physicians in similar contexts phased with a major crisis and as such valuable for future pandemic preparedness.

We have analyzed the material based on discourse psychology and new institutional theory, where we have focused on regulative, normative, and cognitive elements. What we may not have managed to capture with these analytical tools are the interviewees' underlying emotional reactions, which may be interesting to further study.

## Data availability statement

The interview data analyzed during the current study are available from the corresponding author upon reasonable request. Due to the sensitive content in the material, and the Swedish Ethical Review Authority, the data is shared with caution.

## Ethics statement

The study gained ethical approval from the Swedish Ethical Review Authority (2020-02433 dnr:2020-06110). All participants gave their consent to participate both verbally and written.

## Author contributions

EB conducted 26 interviews and wrote most of the background and discussion. MJ and MH conducted the analysis and wrote the result section and discussion and knowledgeable in the theoretical approaches of discourse psychology, and MJ is also knowledgeable in the neo-institutional theory used in this study. FB conducted two interviews, participated with text and in the discussion of the results, and gave valuable comments on the manuscript. All authors contributed to the design of the study. This study is a collaboration by all authors. All authors contributed to the article and approved the submitted version.
