# Supplementary material for: Challenges in preserving the “good doctor” norm: physicians' discourses on changes to the medical logic during the initial wave of the COVID-19 pandemic
Source: Front Psychol. 2023 Jun 8;14:1083047. doi: 10.3389/fpsyg.2023.1083047 (PMC10285475; doi:10.3389/fpsyg.2023.1083047)
Supplement: Supplementary file 1 [file Table_1.DOCX]

**Interview guide 4: Doctors' experiences of working during the Covid-19 pandemic**

| Theme | Question |
| --- | --- |
| Introduction | Leave short info about the study with contact data, tell briefly about your own background, go through consent form, the possibility to cancel, withdraw, then capture the signature |
| Background | - Can you tell us how long you have worked as a doctor and about your specialty, workplace, other tasks (MLA, union manager etc), proportion of clinical work  - What does your private life situation look like? (Children, partner, what does partner work with, larger family) |
| PTSD ”screening” | 1 Do you have any recurring difficult memories or residual psychological discomfort (e.g. unpleasant dreams, intrusive memories, anxiety) from working with the pandemic?  2 Have you made an effort to avoid thinking about or remembering distressing details or feelings related to the event?  3 Have you had any follow-up with management or colleagues to get relief? (or someone at home)  4 Have you avoided talking about the situation because it evokes memories?  5 Have you received/been offered any support or treatment?  *If yes on 1, 2 and 4 but no to follow-up/support/treatment:*  *1) do you feel that you need professional help to process your experiences,*  *2) what kind of help and do you know who they could be and*  *3) Do you know where to turn to get this help/If no – this is an employer's responsibility and refer to your immediate manager.* |
| Experiences of the transition from regular care to pandemic care | - Can you describe your experiences of **the transition** from regular operations to pandemic care?  - How did it affect you? Were you **involved** in the transition? Were your skills utilized? What did you do in the transition? |
| Leadership and organization in the transition | - What was your most important source of **information** during the pandemic? Did this change in the meantime?  - How would you describe **communication** (What was good? Less good? Something you will take with you in the future? ):  Within the collegium of the clinic  Between you in the medical college and your immediate manager during  Between clinics  - How do you expereince that your immediate **manager has handled the department** and the transition during the pandemic?  - How do you feel that the **organization has handled the** pandemic? Exemplify |
| Experiences of being involved in pandemic care | - Can you describe a "**normal" working day** during the pandemic?  - How does it differ from a normal working day before the pandemic? Anything that surprised you? Anything that was better? Something that was worse?  - What do you feel is the **biggest challenge** of working during the pandemic?  - How do you experience working with protective equipment?  - Do you feel any **anxiety or fear** about working with pandemic patients? How do you deal with these fears? What was most important to you in this management? |
| Experience of quality and prioritization during the pandemic | - Can you tell us if your **medical decision-making** was affected during the pandemic? In what way? Did this change over time? Something that was good? Something that was less good?  - What is your experience of **quality of care** and prioritization during the pandemic?  - Has your **contact/relationship** with patients changed in any way?  - How did you work in **the working group on medical decisions and quality of care**? Exemplify. Is there any experience that you will take with you going forward? |
| Experiences of individual existential health and stress of conscience | - Are there any situations that you experienced particularly **stressful** during the pandemic? Can you tell us about these?  - Can you tell us about any situation that you found **ethically challenging**? What did you do then?  - Have you experienced a situation where you had to make difficult decisions and then felt **did not turn out well/right**?  - Have you experienced a situation where you had to make difficult decisions and then felt **was good/right**?  - Is there anything in these situations that you and/or you in the working group will include in the future? |
| Work and life during the pandemic | - How did you experience the opportunity to **combine work and private life** during the pandemic? Did it change over time? Did you have a strategy to simplify balancing work with family?  - How do you feel that the pandemic has **affected you as a person**? And your immediate family? (emotional stress, health). Has it changed over time?  - Is there anything in your personal life that has helped you cope with the work situation during Covid-19 (training, social, nature, support from partners)?  - Have you felt that during the pandemic crisis you had the need to take any drugs or other substances (including alcohol) to cope? Or to get some sleep?  - Have you told anyone this?  - Have you received/been offered any support or treatment?  (*If the person wants help – see PTSD screening*) |
| Contagion | - Have you **contracted Covid-19** while working with corona patients? How did you experience it?  - Have you had symptoms that turned out to be Covid-19 negative?  - How did it feel? And how did that affect your work?  - Have you received support from your nearest manager/hospital in the event of detection of infection?  - How did you experience the reaction from your colleagues? |
| Future | \| - How would you like to sum up your experiences of the Covid-19 pandemic? Feel free to exemplify  - Is there anything you take with you... New insights, thoughts, ideas?  - When the pandemic is over, what do you think will happen then (work and private)?  - Where will you stand in 3 years?  - How do you think the pandemic will affect you and your workplace (short and long term)? \| \| --- \| |

Is there anything that you think we forgot to ask about?

PROBES: support questions to follow up with in general in all themes:

When did you do it?

What did you do then?

Were there more of you who did it?

How did it go?

Do you usually do that?

Does it help why/why not - what works and what doesn't?

Positive/negative of the events?
